# Epidemiology of Trypanosomiasis in Wildlife—Implications for Humans at the Wildlife Interface in Africa

**DOI:** 10.3389/fvets.2021.621699

**Published:** 2021-06-14

**Authors:** Keneth Iceland Kasozi, Gerald Zirintunda, Fred Ssempijja, Bridget Buyinza, Khalid J. Alzahrani, Kevin Matama, Helen N. Nakimbugwe, Luay Alkazmi, David Onanyang, Paul Bogere, Juma John Ochieng, Saher Islam, Wycliff Matovu, David Paul Nalumenya, Gaber El-Saber Batiha, Lawrence Obado Osuwat, Mahmoud Abdelhamid, Tianren Shen, Leonard Omadang, Susan Christina Welburn

**Affiliations:** ^1^Infection Medicine, Deanery of Biomedical Sciences, College of Medicine and Veterinary Medicine, The University of Edinburgh, Scotland, United Kingdom; ^2^School of Medicine, Kabale University, Kabale, Uganda; ^3^Department of Animal Production and Management, Faculty of Agriculture and Animal Sciences, Busitema University Arapai Campus, Soroti, Uganda; ^4^Faculty of Biomedical Sciences, Kampala International University Western Campus, Bushenyi, Uganda; ^5^College of Veterinary Medicine Animal Resources and Biosecurity, Makerere University, Kampala, Uganda; ^6^Department of Clinical Laboratories Sciences, College of Applied Medical Sciences, Taif University, Taif, Saudi Arabia; ^7^School of Pharmacy, Kampala International University Western Campus, Bushenyi, Uganda; ^8^Department of Agriculture, Faculty of Vocational Studies, Kyambogo University, Kampala, Uganda; ^9^Biology Department, Faculty of Applied Sciences, Umm Al-Qura University, Makkah, Saudi Arabia; ^10^Department of Biology, Faculty of Science, Gulu University, Gulu, Uganda; ^11^Faculty of Agriculture and Environmental Science, Muni University, Arua, Uganda; ^12^Department of Biotechnology, Lahore College for Women University, Lahore, Pakistan; ^13^Department of Pharmacology and Therapeutics, Faculty of Veterinary Medicine, Damanhour University, Damanhour, Egypt; ^14^School of Medicine and Health Sciences, Soroti University, Soroti, Uganda; ^15^Department of Parasitology, Faculty of Veterinary Medicine, Aswan University, Aswan, Egypt; ^16^Zhejiang University-University of Edinburgh Institute, Zhejiang University School of Medicine, Zhejiang University, Haining, China

**Keywords:** trypanosomes in wildlife, human-wildlife interactions, wildlife-livestock interactions, human African trypanosomiasis, sleeping sickness, *Trypanosoma brucei gambiense*, *Trypanosoma brucei rhodesiense*

## Abstract

While both human and animal trypanosomiasis continue to present as major human and animal public health constraints globally, detailed analyses of trypanosome wildlife reservoir hosts remain sparse. African animal trypanosomiasis (AAT) affects both livestock and wildlife carrying a significant risk of spillover and cross-transmission of species and strains between populations. Increased human activity together with pressure on land resources is increasing wildlife–livestock–human infections. Increasing proximity between human settlements and grazing lands to wildlife reserves and game parks only serves to exacerbate zoonotic risk. Communities living and maintaining livestock on the fringes of wildlife-rich ecosystems require to have in place methods of vector control for prevention of AAT transmission and for the treatment of their livestock. Major *Trypanosoma* spp. include *Trypanosoma brucei rhodesiense, Trypanosoma brucei gambiense, and Trypanosoma cruzi, pathogenic for humans, and Trypanosoma vivax, Trypanosoma congolense, Trypanosoma evansi, Trypanosoma brucei brucei, Trypanosoma dionisii, Trypanosoma thomasbancrofti, Trypanosma elephantis, Trypanosoma vegrandis, Trypanosoma copemani, Trypanosoma irwini, Trypanosoma copemani, Trypanosoma gilletti, Trypanosoma theileri, Trypanosoma godfreyi, Trypansoma simiae*, and *Trypanosoma (Megatrypanum) pestanai*. Wildlife hosts for the trypansomatidae include subfamilies of Bovinae, Suidae, Pantherinae, Equidae, Alcephinae, Cercopithecinae, Crocodilinae, Pteropodidae, Peramelidae, Sigmodontidae, and Meliphagidae. Wildlife species are generally considered tolerant to trypanosome infection following centuries of coexistence of vectors and wildlife hosts. Tolerance is influenced by age, sex, species, and physiological condition and parasite challenge. Cyclic transmission through *Glossina* species occurs for *T. congolense, T. simiae, T. vivax, T. brucei*, and *T. b. rhodesiense, T. b. gambiense*, and within *Reduviid* bugs for *T. cruzi. T. evansi* is mechanically transmitted, and *T. vixax* is also commonly transmitted by biting flies including tsetse. Wildlife animal species serve as long-term reservoirs of infection, but the delicate acquired balance between trypanotolerance and trypanosome challenge can be disrupted by an increase in challenge and/or the introduction of new more virulent species into the ecosystem. There is a need to protect wildlife, animal, and human populations from the infectious consequences of encroachment to preserve and protect these populations. In this review, we explore the ecology and epidemiology of *Trypanosoma* spp. in wildlife.

## Introduction

The African and American trypanosomiases present significant global health challenge in human, domesticated animal, and wildlife communities. Spillover of parasites from wildlife to domestic livestock and humans and from domestic animal species to wildlife compromises health (1, 2). Most trypanosome infections in wildlife do not cause obvious damage to their host (3, 4), but some wildlife species are highly susceptible to trypanosome infections, including *Rattus nativitatis* and Macleay's rats (*Rattus macleari*) (5).

*Trypanosoma* are primarily transmitted by vectors (6) within which they undergo complex development cycles. Trypanosomes, which develop in the posterior section of the digestive tract in insects, are called stercorarian, for example, *Trypanosoma cruzi* the causal agent of Chagas disease, common in Latin America (7). Salivarian trypanosomes develop in the anterior part of the insect gut tract and include the causal agents of African animal trypanosomiasis (AAT) or nagana and for human African trypanosomiasis (HAT) caused by *Trypansoma brucei rhodesiense* and *Trypansoma brucei gambiense* that are present across Sub-Saharan Africa (8).

Animal trypanosomiasis is endemic in tropical regions of Africa, parts of Asia, and South America (9). *T. brucei s.l., Trypanosoma congolense, Trypanosoma simiae*, and *Trypanosoma uniforme* are transmitted within the tsetse belts of Africa and cannot be transmitted by mechanical vectors (9). *T. vivax* and *Trypansoma evansi* can be transmitted mechanically and occur within and outside tsetse fly-infested zones (10).

## Epidemiology of Animal Trypanosomiasis

Trypanosomiasis is one of the most important diseases affecting livestock, equines, and dogs within the Sub-Saharan region (11, 12). Cross transmission of parasites between livestock and wildlife hosts has been reported, especially in areas in close proximity to game parks and wildlife reserves. Wildlife species can survive within the tsetse belts across the Sub-Saharan region, despite being reservoir hosts for multiple species of trypanosome. The high prevalence of trypanosomiasis within protected areas traditionally has rendered these areas unattractive for livestock keeping and agricultural production (13).

Phylogenetic analysis shows a remarkable complexity of trypanosome species, subspecies, and strains in tsetse flies, human, domestic, and wildlife hosts. Examining the trypanosome species circulating within an ecosystem is a key to identifying the wildlife reservoirs of infection and transmission parameters to other animal hosts, including livestock within the ecosystem (3). Understanding the various trypanosome species harbored in wildlife hosts can guide preventive and control measures of trypanosomiasis in communities living at the livestock–wildlife interface. A wide variety of trypanosome species are circulating among wildlife hosts including *T. brucei s.l., T. congolense, T simiae, T. godfreyi*, and *T. theileri* (3).

### Hosts and Species of Trypanosomes

Apart from *T. cruzi*, present in South America, and *T. theileri*, present worldwide, trypanosomes infect a large number of wild and domestic ungulate species (6). Infection in the wildlife is influenced by species and habitat (12). Wildlife hosts for trypanosomiasis are numerous and include antelope species, warthogs (*Paecocherus aethiopicus*), elephants (*Loxodanta africana*), hippopotamus (*Hippopotamus amphibius*), lions, hyenas, jackals, caracals, and wild ruminants (14–16). Trypanosome species commonly found in wildlife species include *T. vivax, T. brucei s.l., T. congolense*, and *T. evansi* (14). *T. vivax*, a pathogen affecting cattle, has been identified in waterbucks and giraffes, but the strains of *T. vivax* in these two host species were unique (3). Multiple wildlife hosts carry the human-infective zoonotic trypanosome strain *T. b. rhodesiense*, including bushbucks (*Tragelaphus scriptus*), impala (*Aepyceros melampus*), lion (*Panthera leo*), zebra (*Equus quagga boehma*), warthog (*Phacocoerus africanus*), and duiker (*Sylvicapra grimmia*) (12). *Trypanosoma conglense, Trypanosoma simiae*, and *Trypanosoma godfreyi* were identified in Rhinocerus posttranslocation (16).

Infection with trypanosomes can predispose infected animals to other infections (17), and concurrent and opportunistic bacterial infections in wildlife can hasten the onset of clinical trypanosomosis (17).

There are significant associations between taxonomic groups of wildlife hosts and the prevalence of trypanosomiasis. Wildlife hosts from the bovinae group show a high prevalence of trypanosomiasis, especially *T. vivax* and *T. congolense* as well as human infective *T. brucei* (Table 1). Infection is attributed to their grazing habits, taking them into contact with tsetse and other biting flies. Wildlife hosts from the Pantherinae group show a very high prevalence of mixed trypanosome infections, as do those from the Suidae (12). A summary of trypanosome species in wildlife hosts and host taxonomy is shown in Table 1.

**Table 1:** Trypanosome species and taxonomy of wildlife hosts.

**Taxonomic group**	**Wildlife host (scientific name)**	**Trypanosome species**	**References**
Bovidae	Waterbuck *(Kobus ellipsiprymnus)*	*T. vivax, T. congolense, T. brucei, T. evansi*	(12)
Girrafidae	Girraffe (*Giraffa camelopardalis*)	*T. vivax, T. evansi, T. brucei*	(12)
Bovidae	African buffalo (*Syncerus caffer)*	*T. b. rhodesiense, T. congolense, T. brucei*	(12)
	Bushbuck (*Tragelaphus scriptus*)	*T. b. rhodesiense, T. congolense, T. vivax, T. evansi*	(12)
	Greater kudu (*Tragelaphus strepsiceros*)	*T. congolense, T. vivax*	(12)
	Red lechwe (*Kobus leche*)	*T. theileri*	(18)
	Hartebeest (*Alcelaphus buselaphus*)	*T. godfreyi, T. brucei, T. godfreyi*	(18)
	Sable antelope (*Hippotragus niger*)	*T. brucei, T. theileri*	(18)
	African buffalo (*Syncerus caffer*)	*T. theileri, T. godfreyi*	(18)
	Eland (*Taurotragus derbianus)*	*T. vivax, T. congolense, T. brucei*	(12)
	Impala (*Impala impala*)	*T. godfreyi, T. brucei*	(18)
Elephantidae	Elephant (*Loxodanta africana*)	*T. vivax, T. congolense, T. evansi, T. elephantis*	(19)
Hippopotamidae	Hippopotamus (*Hippopotamus amphibius*)	*T. vivax, T. brucei, T. evansi, T. congolense*	(20)
Suidae	Warthog (*Phacocoerus africanus)*	*T. b. rhodesiense, T. vivax, T. congolense, T. evansi*	(20)
	Warthog (*Phacochoerus africanus*)	*T. godfreyi, T. brucei, T. simiae*	(18)
	Feral pig (*Sus scrofa*)	*T. evansi and T. cruzi*	(21)
Pantherinae	Lion (*Panthera leo*)	*T. brucei, T. evansi, T. congolense, T. congolense*	(12, 18)
	Leopard (*Panthera pardus)*	*T. brucei, T. congolense, T. evansi*	(12)
Equidae	Zebra (*Equus quagga boehma*)	*T. b. rhodesiense*	(12)
Cephalophinae	Common duiker (*Sylvicapra grimmia*)	*T. b. rhodesiense, T. vivax, T. congolense*	(12)
Aepycerotinae	Impala (*Aepyceros melampus*)	*T. b. rhodesiense, T. congolense, T. evansi*	(20)
Rhinocerotidae	Rhino (*Diceros bicornis)*	*T. brucei*	(20)
Alcephinae	Wildebeest (*Connochaetes taurinus cooksoni)*	*T. brucei, T. congolense, T. vivax*	(20)
	Hartebeest (*Alcephalus buselaphus*)	*T. evansi, T. brucei*	(20)
Hyaenidae	Hyena (*Hyaena hyaena)*	*T. evansi, T. congolense*	(12)
Cercopithecinae	Vervet monkey *(Cercopithecus species)*	*T. gambiense*	(12)
	Baboon (*Papio cynocephalus)*	*T. congolense*	(12)
Crocodilinae	Crocodile (*Crocodylus niloticus*)	*T. vivax*	(12)
Hippotraginae	Roan antelope (*Hippotraggus equinus)*	*T. vivax, T. congolense*	(12)
Pteropodidae	Megabat/fruit bat (*Chiroptera*)	*Trypanosoma dionisii, T. cruzi*	(22, 23)
Phalangeridae	Brushtail possum (*Trichosurus vulpecula*)	*Trypanosoma* spp.	(17)
Muridae	Brush-tailed rabbit-rat (*Conilurus penicillatus*)	*Trypanosoma* spp.	(5)
Potoroidae	Brush-tailed bettong (*Bettongia penicillata*)	*T. vegrandis, T. copemani*	(24)
Dasyuridae	Northern quoll (*Dasyurus hallucatus*)	*Trypanosoma* spp.	(5)
Peramelidae	Northern brown bandicoot (*Isoodon macrourus*)	*Trypanosoma* spp.	(5)
Phascolarctidae	Koalas (*Phascolarctos cinereus*)	*Trypanosoma irwini, T. copemani*	(25)
	Koalas (*Phascolarctos cinereus*)	*T. gilletti*	(25)
Cervidae	Marsh deer (*Blastocerus dichotomus*)	*Trypanosoma theileri, T. evansi*	(26)
Canidae	African wild dog (*Lycaon pictus*)	*T. godfreyi*	(18)
Potoroidae	Boodie (*Bettongia lesueur*)	*Trypanosoma spp*.	(27)
Tayassuidae	White-lipped peccary (*Tayassu pecari*)	*Trypanosoma evansi and Trypanosoma cruzi*	(21)
Mustelidae	Wild European badger (*Meles meles*)	*Trypanosoma (Megatrypanum) pestanai*	(28)
Meliphagidae	Regent honeyeater (*Anthochaera phrygia*)	*Trypanosoma thomasbancrofti*	(29)

### Transmission to Wildlife

Transmission of trypanosome species is generally by biting vectors including *Tsetse flies, Tabanids, Stomoxys, Heamatopota*, and *Hippobosca* (15, 30, 31). Infection in carnivores is additionally from consumption of infected meat as documented in the Felidae and Canidae (31, 32). *Desmodus rotundus* (Vampire bats) also transmit trypanosomiasis (32).

Trypanosomes engage in two patterns of transmissions: Cyclical transmission in which trypanosomes undergo active multiplication within the vectors (tsetse flies) as is common for *T. congolense, T. simiae, T. vivax, T. brucei*, and the human infective trypanosome species (*T. rhodesiense and T. gambiense*); and mechanical transmission through tsetse and alternative vectors including biting flies of the Tabanidae family (horse flies) as well as Stomoxys species. *T. evansi* and *T. vivax* can be mechanically transmitted (33).

### Distribution of Reservoir Hosts

Preservation of natural resources including game parks and game reserves has led to an expansion of wildlife populations that serve as reservoirs for AAT and HAT (34). Human encroachment on the game parks and forests has increased AAT transmission between wildlife and domestic livestock, due to increased tsetse–human and tsetse–livestock contacts (34). The distribution of host populations within these areas determine vector and parasite survival. Wildlife hosts including monkeys and warthogs live in less restrictive habitats, unattractive to poachers with limited trophy hunting leading to increased reservoir host multiplication rates. They are widely distributed in the ecological environment and are favorable reservoirs for multiple trypanosome species due to their availability to vectors. Crocodiles and hippopotamus are limited to specific habitats, limiting access of vectors.

### Distribution of Tsetse Flies

Trypanosomiasis affects one-third of Africa's land mass (35–37). Tsetse are found across most of West, Eastern, Central, and Southern Africa (38). The different species and subspecies of tsetse are shown in Table 2. Tsetse populations require moderate temperatures (23–25°C), high relative humidity (75–90%) with weak saturation deficit and shade (47–49). Temperatures above 34.1°C limit survival of tsetse and trypanosomes (35).

**Table 2:** Tsetse species, geographical distribution, and wildlife spp. affected by trypanosomiasis.

**Subgenus**	**Glossina species**	**Glossina subspecies**	**Country**	**Wildlife animal spp**.	**References**
*Nemorrhina (Palpalis)*	*Glossina palpalis*	*G. p. palpalis*	Nigeria, Angola Cameroon,	Bushbuck, primates, warthogs	(36)
	*G. tachinoides*		Nigeria		(37)
		*G. p. gambiesis*	Gambia, Senegal, Republic of Guinea	Baboons, monkeys, chimps	(39, 40)
	*G. fuscipes*	*G. f. fuscipes*	Uganda, Sudan, Ethiopia, Kenya, DRC	Buffaloes, antelopes	(41)
		*G. f. martini*	Uganda, Tanzania, DRC	Buffaloes	(42)
		*G. f. quanzensis*	Uganda, Tanzania	Buffaloes, antelopes	(42)
	*G. pallicera*	*G. p. pallicera*	Cameroon, Ivory coast, Liberia	Antelopes	(43–45)
		*G. p. newsteadi*	DRC	Lions, leopards	(46)
	*G. tachinoides*		Nigeria, Ghana, Cameroon	Buffaloes, lions, buffaloes	(46)
	*G. caliginea*		Nigeria, Congo Brazaville	Cheetah, lions, leopards	(46)
*Glossina (morsitans)*	*G. morsitans*	*G. m. morsitans*	Nigeria, Uganda, Tanzania, Zambia	Buffaloes, rhinoceros, antelopes	(46)
		*G. m. submorsitans*	Uganda, Tanzania	Buffaloes, bushbuck, antelopes	(46)
		*G. m. centralis*	Uganda, Tanzania	Buffaloes, bushbucks, antelopes	(46)
	*G. swynnertoni*		Nigeria, Congo Brazaville	Lions, cheetahs	(46)
	*G. longipalpis*		Ivory Coast, Senegal, Mali	Buffaloes, lions	(46)
	*G. pallipides*		Ethiopia, DRC, Uganda, Kenya, Zambia, Tanzania	Buffaloes, lions, antelopes	(46)
	*G. austeni*		Kenya, Tanzania, Mozambique	Bushbucks, antelopes, lions	(46)
	*G. vanhoofi*		DRC	Lions	(46)
	*G. tabanformis*		Nigeria, DRC	Buffaloes, lions	(46)
	*G. severini*		DRC	Lions, bushbucks	(46)
	*G. schwetzi*		Togo. Congo Brazaville	Wild ruminants	(46)
	*G. nigrofusca*		Ivory Coast, Nigeria, CAR, DRC	Elephants, lions, monkeys	(46)
	*G. nashi*		Cameroon, Nigeria, Togo	Monkeys, baboons, wild cats	(46)
	*G. medicorum*		Ghana, Gambia, Nigeria	Lions, buffaloes	(46)
	*G. longipennis*		Tanzania, Sudan, Kenya	Antelopes, bushbucks, lions	(46)
	*G. hanningtoni*		Nigeria, Cameroon, Gambia	Bushbucks, buffaloes	(46)
	*G. fuscipleuris*		CAR, DRC Cameroon	Lions, bushbucks	(46)
	*G. brevipalpis*		Kenya, DRC, Tanzania	Buffaloes, antelopes	(46)

### Food Source—Activity and Migration

Differences in wildlife food sources, particularly for wild bovidae, influence their exposure to trypanosomiasis. Among ruminant wildlife hosts, browsers are more at risk of trypanosomiasis compared with grazers; semi-browsers are moderately susceptible. Eland, Waterbuck, Kudu, and Bushbuck are more heavily infected, associated with their preference for inhabiting thickets during tsetse feeding hours, predisposing them to more bites (13, 18).

Diurnal wildlife hosts are more susceptible to trypanosome infection compared with the nocturnal species. Warthogs are most active in the morning and late afternoon and show high infection rates for trypanosomiasis in correlation to vector feeding hours (20). Lions are more infected in areas of high tsetse challenge than low challenge (50). The movement of large numbers of animals within wildlife ecosystems also influences infection. Animals will migrate in the dry season toward water sources that are also tsetse habitats (20).

### Pathogenesis of Trypanosomosis

Trypanosome infection leads to erythrophagocytosis and heme catabolism resulting in iron accumulation in tissues and hyperbilirubinemia, liver dysfunction, and multiple organ failure (51). At necropsy, atrophy of body fats, pulmonary edema, hepatomegaly, lymphadenopathy, and hemorrhages are observed. Trypanosomes are found in tissues and body organs, and enlarged periarteriolar sheaths have been observed in wildlife (52).

### Clinical Signs

Trypanosomiasis is a chronic progressive disease, and clinical signs may become obvious in advanced stages of the disease (53). Bovines affected by *T. vivax* present with severe anemia, lethargy, photophobia, lacrimation, and inappetence (17, 54, 55); pyrexia fluctuates with the fluctuating parasitemia. Leukopenia, thrombocytopenia, and degenerative and inflammatory lesions are observed in most organs (56). Body condition scores deteriorates gradually, and animals are dehydrated and debilitated before death. Superficial lymph nodes are enlarged and conspicuous. Corneal opacity may be observed with lacrimation (57). Young animals are stunted even with proper feeding, and productivity is impaired by abortions (32, 38). Animals may show localized or generalized edema (58). Except for *T. equiperdum*, other *Trypanosoma* species include similar clinical signs, but variations in intensity may happen in the various species. *Trypanosoma brucei* s.l. infection shows limited clinical signs in bovines of indigenous species but is highly pathogenic in exotic species (59).

### Diagnosis of Trypanosomiasis

Trypanosomiasis is characterized by the intermittent presence of parasites in the blood and intermittent fever (54). Parasitological examinations are relatively sensitive during the acute phase of the disease. Wet blood film examination is used to detect the presence of motile trypomastigotes but has low sensitivity. Blood is taken from the tail or ear veins (55). Fluorescence microscopy can improve sensitivity for thin and thick smears (60). Parasitic concentration by centrifugation and examination of the buffy coat is more sensitive than the wet and thick smears (61). Dark ground or phase-contrast microscopy increases sensitivity at low parasitemia (62). Anion exchange chromatography is also sometimes deployed for detecting low parasitemia (54, 63, 64).

Molecular tests and serological tests are more sensitive than the usual parasitological tests for *Trypanosoma* (3, 63–68). Common serological tests include CFT, ELISA, and IFAT, while the common molecular tests are PCR, LOOP/LAMP, and LFA. Low-flow assay (LFA) is cheaper with 96.3% sensitivity and 93.9% specificity (69). Approved tests for AAT are shown in Table 3.

**Table 3:** Approved laboratory tests for trypanosomiasis according to OIE (70).

**Test criteria**	**Objective**	**Methods**	**References**
Clinical signs	Categorize presentation	Observations	(32, 53, 71)
Microscopy	Direct examination	Wet blood films	(72)
		Thick blood films	(60, 61)
		Thin blood smear films	(72)
	Parasitic concentration	Microhematocrit centrifugation technique	(61)
		Dark ground or phase-contrast buffy coat technique	(62)
		Anion exchange technique	(73–75)
	Cultivation technique	Animal inoculation	(76–78)
Molecular detection	Antigen assays	Trypanosome antigen detection assays	(79)
	Trypanosome DNA	Monospecific PCR	(80)
		Multi-specific PCR	(81, 82)
		LOOP	(68)
		LFA	(69, 83)
Serology	Antibodies	Indirect immunofluorescence test	(84)
		IgG antibody ELISA	(85)
		IgG antibody detection	(86, 87)

## Trypanosomes in Wildlife

The majority of trypanosome species require multiple obligatory hosts to complete their life cycles (heteroxenous), and the transmission of the parasites is mainly via hematophagous invertebrate vectors (2, 88). Trypanosomes are found in blood and tissues; blood-borne protozoan trypanosomes (*Trypanosoma vegrandis*) have been identified in wild animals including, but not limited to, the northern brown bandicoot (*Isoodon macrourus*), common brushtail possum (*Trichosurus vulpecula*), northern quoll (*Dasyurus hallucatus*), and brush-tailed rabbit-rat (*Conilurus penicillatus*) (5). *Trypanosoma cruzi, Trypanosoma dionisii*, and an insect trypanosome (*Blastocrithidia*) have been found to infect bats and other mammalian wildlife in Europe and South America (22). Bats, possums, and rats act as reservoirs of trypanosomes for domestic and wild animals, as well as humans (5, 22). *T. cruzi* (Chagas) has been identified in kidney tissue, heart muscle, and blood, urine, and peritoneal fluid of wild spp. including foxes, opossums, raccoons, and striped skunks (89, 90), and parasites can be transmitted from animal-to-animal by contamination of animal wounds with blood, urine, and peritoneal fluid (89, 90).

### Trypanosome–Host Relationships

Hosts are classified according to their role as a definitive host [if the mature sexual stage(s) of the parasite occurs within them] or intermediate hosts when the more mature sexual stages of the parasite only aid part of the life cycle (91). Transfer (paratenic) hosts are not vital for the completion of parasitic development cycles but maintain the parasite before it reaches the obligatory/definitive host (92, 93). Invertebrates (vectors) acting as hosts and carriers of parasites facilitate the completion of parasitic life cycles by transmitting parasites (94–96).

Trypanosomes can infect many hosts, transmitted by hematophagous insect vectors mainly the tsetse fly and triatomid kissing bugs (subfamily: Reduviidae) (13, 97). Salivarian trypanosomes, *Trypanosoma brucei, T. rhodesiense, T. equiperdum, T. vivax*, and *T. congolense* are transmitted in tsetse fly (*Glossina* spp.) saliva to the host spp. Hosts are as follows: *T. brucei* s.l. (domestic mammals and humans), *T. vivax* (ruminants, horses, and camels), *T. equiperdum* (equines), *T. simiae* (pigs), and *T. congolense* (dogs and cats) causing *T. evansi* (domestic mammals), and other numerous wildlife hosts such as monkeys, guinea pigs, rabbits, rats, etc., are also affected (91, 98). Stercorian trypanosomes are transmitted through the fecal matter of the insects (*Triatominae*, e.g., *Triatoma infestans*) to host skin where they gain access to tissues. Other vectors of transmission for stercorians include Tabanid flies, stable flies, ticks, and mosquitoes. Stercorians include *Trypanosoma cruzi, T. lewisi, T. melophagium, T. nabiasi, T. rangeli, T. theileri, T. theodori, T. lewisi, T. cruzi*, bat spp. (*Trypanosoma cruzi marinkellei, Trypanosoma dionisii, Trypanosoma erneyi, Trypanosoma livingstonei*, and *Trypanosoma wauwau*), and others: *Trypanosoma conorhini* and *Trypanosoma rangeli* (94, 95, 98, 99). Among wildlife, *T. cruzi* is found in armadillos, dogs, possums, foxes, bats, raccoons, and striped skunks (5, 22, 89, 90). In addition, *T. melophagum* and *T. theileri* are found in Europe infecting cattle, buffaloes, and antelopes (98).

### Trypanotolerance

Trypanotolerant animals show a few clinical signs (96, 97), and trypanosomes are able to evade the host immune responses (100). Trypomastigotes penetrate a variety of host cell types and multiply intracellularly as amastigotes—which eventually infect host cells and differentiate into BFT, which eventually invade the lymphatic and circulatory systems (50, 101). *Trypanosoma* cell membranes are covered with dense variable surface glycoprotein (VSG) homodimers—immunodominant antigens that trigger infected host B- and Th-cell-mediated immune responses. Over 1,000 different VSG genes and pseudogenes are present in the trypanosome genome that can undergo segmental gene recombination to encode an estimated 10,000 different VSG surface coats. High antigenic variability in VSG molecules hinders vaccine development (102).

Wildlife, while generally immunotolerant to trypanosomes (103) can, however, develop clinical trypanosomiasis (104) and show varying levels of trypanotolerance among species (105, 106). Trypanotolerance is influenced by multiple host intrinsic and extrinsic factors and can develop from previous exposure (106). Intrinsic factors include age, sex, species, physiological state, and state of nutrition, while extrinsic factors include temperature, humidity, nature of vegetation, and the nature of wildlife communities (107).

A study in the Serengeti National Park, Tanzania, showed age-dependent infection with *T. congolense* in lions (*Panthera leo*); however, the same animals appeared to have developed cross-immunity following infections to multiple trypanosome species including *T. brucei rhodesiense* (101). Lions are exposed to high challenge from trypanosomes, both from tsetse and also from infected prey, becoming exposed to extremely high numbers of VSGs. This work suggests that animals within a closed exosystem can control infections and, in this case, eliminate the human infective parasite *T. b. rhodesiense* from the infection pool.

### Development of Resistance

**Figure 1:**
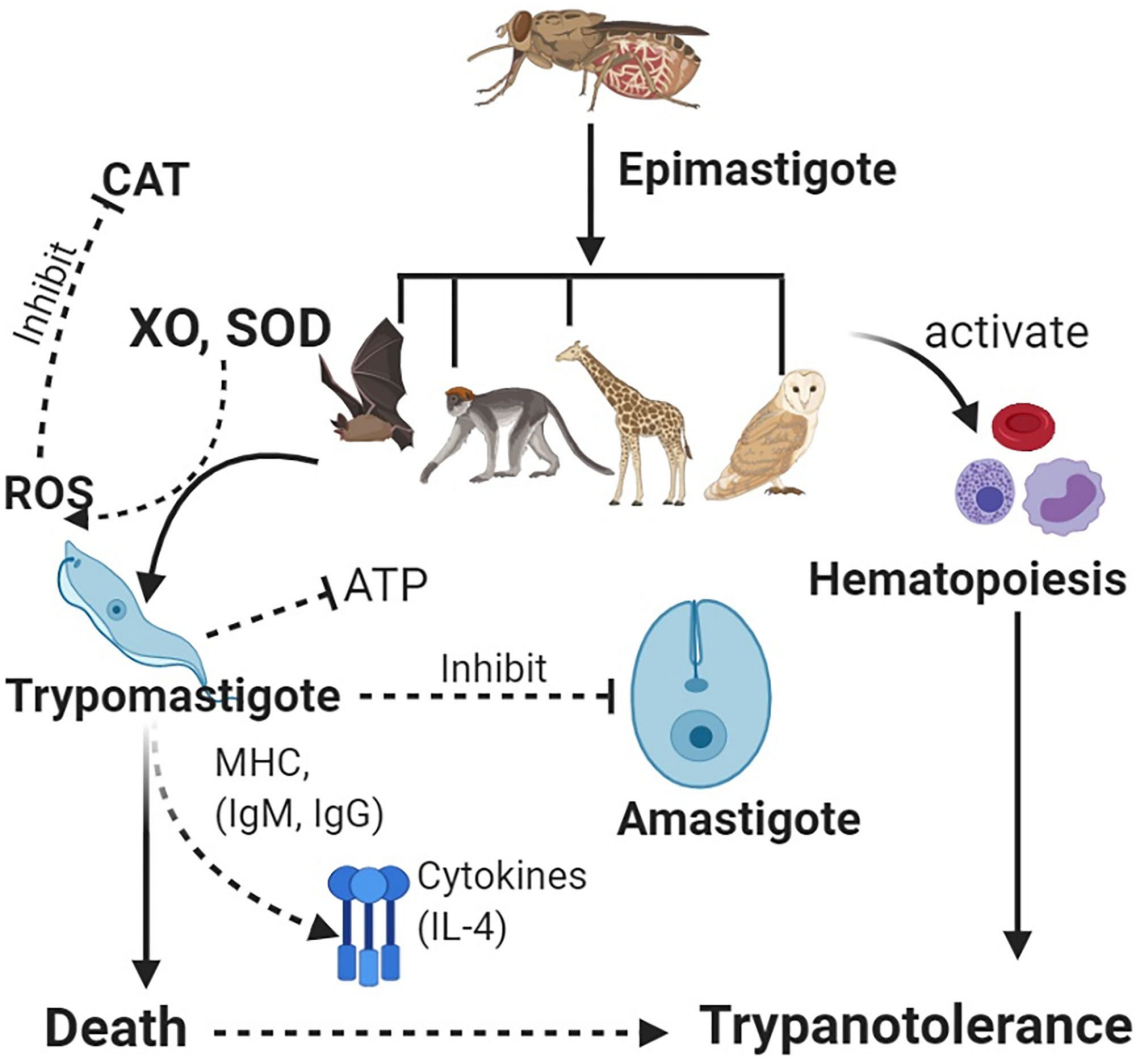
Vectors carrying epimastigotes infect wildlife host species. Epimastigotes transform into trypomastigotes in blood. Host enzymes [e.g., xanthine oxidase (XO) and superoxide dismutase (SOD)] increase production of reactive oxygen species (ROS) such as hydrogen peroxide, which helps clear the parasites, while antioxidant enzymes such as catalase (CAT) lead to increased parasitemia. Immune system activation leads to production of antibodies and cytokines, which neutralize trypanosomes. Inhibition of glycolysis (ATP) leads to deprivation of energy in the parasite for cellular activities. Furthermore, host defenses activate the innate immunity leading to hematopoiesis and an increase in blood cells, thus, leading to trypanotolerance. Wildlife host species subsequently get infected with trypanosomes but rarely succumb to infection.

Trypanotolerance can enable the regulation of parasite levels in the blood (parasitemia) and body tissues. Resistance in wildlife species has been associated with serum xanthine oxidase and catalase and other trypanolytic molecules (108, 109). Stress can affect trypanotolerance in wildlife (71). Trypanotolerance has been investigated in mice and cattle, although these have differences in immune response, i.e., more pronounced B-cell activation in mice than in cattle (50). In cattle, antibody (IgG and IgM) and complement activity against parasitic VSGs leads to protection (110), through antigenic neutralization and IL-4 production (111) (Figure 1).

In Cape buffalo, resistance has been associated with non-specific trypanocidal activity in serum, which helps to lower the parasitemia following action of xanthine oxidase (XO), which generates reactive oxygen species (ROS). Since trypanosomes cannot metabolize XO, this cripples parasitic binding and endocytosis (111), trypanosomes are starved of ATP, and death follows (Figure 1). An increase in catalase activity is associated with increased parasitemia (112). Some wildlife spp. including the black rhinoceros have significant deficiencies for ATP, catalase, and glycolysis enzymes (conditions that favor trypanosome parasitemia) leading to adaptive evolutionary changes, which help to protect the animals against the parasites (113).

Wild animals show varying levels of trypanotolerance; the Thomson's gazelle, dikdik, blue forest duiker, jackal, bat-eared fox, ant bear, hyrax, serval, and monkey are all susceptible to *T. rhodesiense* and *T. brucei*, whereas the common duiker, eland, bohor reedbuck, spotted hyena, oribi, bushbuck, impala, warthog, bushpig, porcupine, and baboon are considered resistant (or less susceptible) to *T. b. rhodesiense* and *T. brucei* infection (114, 115). Animals most susceptible are usually found in areas of high tsetse challenge, while those least susceptible (resistant) animals within the population may have acquired resistance through low exposure and challenge over time (116).

The clinical course of trypanosomiasis has been examined in native African buffalo (*Syncerus caffer*), oryxes (*Oryx beisa*), eland (*Taurotragus oryx*), and waterbuck (*Kobus defassa*) following infection with *T. congolense, T. vivax, or T. brucei*. These animals showed resistance in the form of negligible parasitemia and minimal anemia (115). Wild and domestic animals have been observed to develop resistance to trypanosomiasis after being subjected to prolonged continuous trypanosome infections (117, 118), and as previously mentioned, indigenous bovines are resistant to *T. brucei* within endemic foci in Uganda (114, 115).

## Trypanosomiasis at the Wildlife, Domestic Animal Interface

### Diversity of Trypanosomes in Wildlife

Multiple *Trypanosoma* species and genotypes contribute to a large reservoir of parasite diversity. This presents major problems in the management of trypanosomiasis at the wildlife–domestic animal interface, with the risk of virulent strains emerging that impact both wildlife and domestic populations (5). A review of Australian animal trypanosomes found a huge variety of parasites: *T. pteropi, T. thylacis, T. hipposideri, T. binneyi, T. irwini, T. copemani, T. gilletti, T. vegrandis, T. lewisi, T. melophagium, T. theileri, T. nabiasi, T. evansi, T. cruzi, T. pteropi, T. hipposideri, T. binneyi, T. thylacis, T. copemani, T. Irwin*, and *T. gilleti*. Such biodiversity may have negative impacts on the wildlife and national parks, and is associated with biosecurity concerns (88, 119). Newly identified genotypes of wildlife animals include *Trypanosoma vegrandis G6* and *T. vegrandis G3* (5). Furthermore, a study in bats found three *Trypanosoma* spp. (*T. cruzi, T. dionisii*, and *Blastocrithidia* spp.) (22) demonstrating the great diversity in several wildlife species.

### Infection at the Wildlife, Domestic Animal Interface

Climate change, population pressure, and incentives to end poverty through farming have forced humans to encroach into land previously occupied by wildlife (108, 120). Human encroachment in protected zones runs the risk of parasite transmission from wildlife to domestic animals and zoonotic transmission (108, 109, 121). Synanthropic zoonotic infections are spread from livestock to humans, and exoanthropic infections are spread by wildlife and feral animals to humans—contributing to the increasing gene pool of anthroponoses (98). The cross-species (interspecies) transmission, also known as host jump or spillover, means the potential of an external parasite to invade a new host and infect them to ultimately spread to the whole population of the new host. About 63% of host jumps are responsible for interspecies diseases (109, 122, 123).

Endemic zones are created by encroaching on places of game parks. This has caused a wildlife and livestock interface, and development of the trypanosome parasite reservoirs (117). Wildlife is often implicated as the reservoir of parasites especially trypanosomes (37, 124, 125). It is common for the high incidences of trypanosomiasis in the wildlife to spillover to the domestic cycle in the tsetse fly-infested zones (71). Domestic animals pose a risk to wildlife, particularly the great apes, especially if the domestic carriers are present, for example, cattle and dogs (32).

Infection with *T. b. rhodesiense* is common in communities living proximal to, or that are dependent on, wildlife or eco-tourism (126, 127). In the Luangwa Valley, Zambia, considerable efforts are made to keep domestic animals away from the national park, for biosecurity of livestock keepers, the national parks, and game conservancies (128). In Zambia, HAT infections have been associated with young children attending school and older women demonstrating in the homestead (129, 130).

A study in Australia failed to find *T. cruzi* and *T. evansi* in native domestic and wildlife animals; however, a spillover for exotic *Trypanosoma* spp. was expected that would affect humans, domestic, and wild animals (88). Wildlife (*Clyomys laticeps, Thrichomys pachyurus*, and *Oecomys mamorae)* can be reservoir hosts for *Trypanosoma* spp., e.g., *T. evansi* and *T. cruzi* that could infect humans and other wildlife populations without affecting the rodent spp. (131).

### Factors That Could Influence Trypanosomiasis Epidemiology in Wildlife Areas

Host species that are widely distributed and with fewer restrictions on habitat have proved to be of more importance to trypanosome diversity due to their unlimited breeding potential and less risk for poaching and trophy hunting. Such hosts include warthogs, bushbucks, kudus, buffaloes, and giraffes (20). More habitat-restricted host species have minimal contribution to the epidemiology of trypanosomiasis in wildlife communities due to their relative safety from trypanosome vectors like Glossina and Tabanids, e.g., crocodiles and hippopotamus.

Differences in feeding patterns of the trypanosome hosts influence the distribution of trypanosomiasis in wildlife areas. Certain preferences maintained by certain hosts like bovines (bushbuck, waterbuck, eland, kudu, etc.) to bushy areas and thickets have predisposed them to higher exposure rates to tsetse and other biting flies, intensifying the spread of trypanosomes (20). Trypanosome diversity among the host species has facilitated the cross-transmission of various trypanosome strains and variants among the hosts, increasing infection rates among wildlife communities at both clinical and subclinical levels. For example, the discovery of three different variants of *T. vivax* in three different host species including a buffalo, a waterbuck, and a giraffe not similar to any published strain, demonstrating genetic diversity, provides insights on pathogen epidemiology (3).

Furthermore, the introduction of new host species from a different geographical location into a wildlife reserve can greatly influence the trypanosome species diversity in a wildlife community. This way, new variants and species of trypanosomes are spread from one host to another by tsetse and other biting flies, resulting in devastating effects on wildlife health and livestock health in the area, for example, the discovery of *T. melophagium, T. nabasi, T. theieri*, and *T. evansi* in Australia following the introduction of various mammals into their wildlife populations, which had a great impact on the native marsupials (9).

### Wildlife Encroachment and the Epidemiology of Trypanosomiasis

Encroachment to wildlife and the increasing human and livestock density as well as the altered patterns in land use are key parameters that govern the transmission of trypanosomes (132). It is expected that understanding how the encroachment to wildlife affects the epidemiology of trypanosomes will inspire practical approaches to improve the understanding of epidemiological characteristics of trypanosome transmission in the context of ecological factors. Encroachment to wilderness areas of Africa increases the epidemiology of trypanosomes, hence, increasing the transmission of HAT. Primarily, wildlife are trypanosome reservoirs; however, growing human and livestock numbers around or in wildlife areas increase the significance of livestock in the transmission cycles thereby increasing the epidemiology of trypanosomes pertinent to human health (132).

Understanding undercurrents associated with the transmission of trypanosomes and in relationship with the encroachment of livestock and humans to wildlife areas are vital to developing robust control measures. This would help identify important parameters on host distributions, tsetse populations, epidemiology of trypanosomes, infection and mortality rates, the significance of livestock, humans, and livestock as hosts in wildlife areas, hence, promoting the progress of models to help in the evaluation and application of control measures (132). Parameters that determine the dynamics in encroachment levels to wildlife areas such as protected area, wildlife density, livestock density, human density, and location according to space and time, would help determine the foci of HAT. This is important since increased human and livestock populations and their distribution may lead to land-use pattern changes in fragmented tsetse habitats, and this inevitably affects the distribution of wildlife species. These ultimately result in increased tsetse abundance and distribution, host selection, and tsetse mortality, which are indicators of increased vector competence (132). Host population density is a key factor in determining the dynamics of tsetse populations, i.e., a decline in host density through encroachment to wildlife areas influences tsetse population changes in space and time. This, in turn, influences the transmission of trypanosomes, and although low host density decreases trypanosome transmission as a consequence of tsetse mortality, the low host density may also be associated with an increased level of trypanosome transmission arising from the hungrier flies that bite humans through increased host-seeking efficiency of tsetse flies (133).

## Infection Control at the Wildlife–Livestock–Human Interface

While communities fail to understand the value of wildlife ecosystems, continued wildlife–human conflict presents an increased risk of infection spillover to humans (18, 118, 134, 135). Game parks, the natural habitats for tsetse species, pose risks to livestock and human populations (136, 137), and parasitic infestations among the livestock and humans equally pose a risk to wildlife. Wildlife reservoirs make approaches of trypanosomosis control at the wildlife–livestock–human interface more complicated (30, 31). There is a need to limit the interaction between livestock and wildlife by preventing encroachment into wildlife-protected zones. Mitigation of risk to prevent trypanosome (and other) infections circulating among livestock and wildlife demands a holistic One Health approach (108, 138, 139). Top–down, approaches, shooting games and radical bush clearing, and insecticide spraying in protected zones are neither practical nor acceptable (140). Stakeholder and community-derived solutions are likely to be sustainable options to explore. Approaches to infection control require to be nuanced in these zones, with communication, education, and interventions embedded within the affected communities. Integrated insect control approaches including the use of insecticide-impregnated targets can protect livestock and game (141). Application of insecticides to cattle, using livestock as live baits, can offer sustainable solutions (138, 142, 143); however, challenges remain on the sustainability of this approach especially in low–middle income countries (LMICs). Insecticides are a reliable method for tsetse control and can be improved by deploying an integrated insecticide approach (139, 141). Routine prophylaxis among livestock can protect livestock and offer collateral benefits for humans and wildlife (144, 145). There is also a need to limit the interaction between livestock and wildlife by stopping encroaching on gazetted wildlife zones to lower the trypanosome prevalence in domesticated livestock (108).

Animal and Human Health for the Environment and Development (AHEAD), launched in 2003, comprises a One Health team of socioeconomic scientists, ecobiologists, veterinarians, agriculturalists, wildlife, and public health specialists that address issues at the wildlife, human, and domestic livestock interface. This includes efforts to monitor parasitic diversity in wildlife species to assist in the strengthening of disease surveillance in LMICs (146). Management and communication with regard to wildlife is key to the One Health approach; in pastoral communities, retaliatory persecution through poisonings of predatory wildlife continues to challenge conservation efforts (147). Conflicts associated with competition for natural resources between livestock-keeping communities and wildlife can be mitigated by a combination of communication and control strategies to promote peaceful coexistence of wildlife and humans as promoted by AHEAD.

### Management of Spillover of Trypanosomiasis Among the Human–Wildlife Areas

Communities in the wildlife zones sometimes agree on coexistence with wildlife and the creation of buffer zones (148). However, the coexistence of human communities and wildlife poses risks of outbreaks of various zoonoses (149). In the gazetted wildlife zones, there should be no mixing of domestic animals with wild trypanosomiasis reservoirs. Proper fencing can be used to control the spillover in wildlife borders (150) as part of the integrated trypanosomiasis control strategy. Restrictive models need to be developed by engaging the communities such that they understand these objectives (151). There is a need for legislation on fencing to have restricted movement of livestock and wildlife. The laws need to address the challenges of wildlife biodiversity (152) and penalize encroachers and poachers. This requires an appropriate land tenure system and robust enforcement teams.

Renowned trypanosome hosts like bats need to be removed from urban centers and human dwellings. Bats also live in human dwellings in ceilings and other dark points. African bats are hosts of trypanosomes (153). It is not known whether the African bats are associated with virulent chronic and acute human African trypanosomiasis. However, bats are associated with trypanosomes in central and South America (22, 154, 155).

Research on possible vaccine candidates has not broken through despite emphasis on the VSG pathway (102, 156). This, however, does not translate that research on trypanosome vaccines has reached a dead end since in all the failures, better innovations can transform science to improve understanding in this field (157, 158). This is important since drugs that are used to treat domestic animals have been used to treat wildlife with success (159, 160), although this has not been done against infections with the zoonotic trypanosomes.

Wildlife are usually in contact with insects other than the tsetse flies. It is known that lice can transmit *T. cruzi* (161). The possibility of lice transmitting the zoonotic trypanosomes is not known. Zoonotic trypanosomes have been found in fleas (162), and it is speculated that fleas may transmit trypanosomes among wildlife (163–165). The possible transmission of trypanosomes by other biting arthropods among wildlife needs further investigation. The possibility of transmission along the food chains for carnivores needs further investigation. Wild Canidae that feed on fresh blood from trypanosomiasis reservoirs may acquire infections from the fresh blood. No studies have ever been proposed among at-risk carnivores.

There is a need to study the interactions of trypanosomes with other blood parasites. Trypanosomes interact with babesia especially to worsen the stress conditions of translocation (166–168). The effect of trypanosomes on the immune system likely predisposes the animals to opportunistic infections and tick-borne diseases. The presence of other parasites is possibly a contributing factor to the trypanosomiasis spillover.

## Conclusion

Trypanosomiasis continues to be a major global challenge, particularly so at the wildlife–domestic livestock interface. Multiple wildlife species serve as maintenance hosts promoting infections at the livestock–wildlife interface. There is a high risk of infection spillover from game parks and conservation areas where parasites and vectors are concentrated in high numbers, and domestic livestock pose risks to wildlife-protected species. The basis of trypanotolerance in wildlife species is not well-understood. The wide genetic diversity exhibited by *Trypanosoma* spp. is a challenge, both exacerbating the risk of increased virulence and making the development of a vaccine unlikely. One Health strategies that are community and environmentally friendly are needed to support stakeholders to mitigate risk. There is a need to strengthen trypanosomiasis research, particularly in LMICs, especially at the human–domestic–wildlife interface to prevent cross-species infection.

## Author Contributions

KIK and SCW conceptualized the study, designed the study, and analyzed and interpreted the data. KIK, GZ, FS, BB, KJA, KM, HNN, LA, DO, PB, JJO, SI, WM, DPN, GESB, LOO, TS, MA, LO, and SCW collected the data. KIK wrote initial draft while SCW critically reviewed it for intellectual content. All authors approved the manuscript for publication and remain in agreement on all aspects of the work.

## Conflict of Interest

The authors declare that the research was conducted in the absence of any commercial or financial relationships that could be construed as a potential conflict of interest.
